# Detection of Epstein-Barr virus encoded small RNA genes in oral squamous cell carcinoma and non-cancerous oral cavity samples

**DOI:** 10.1186/s12903-021-01867-8

**Published:** 2021-10-07

**Authors:** Arghavan Zebardast, Yousef Yahyapour, Maryam Seyed Majidi, Mohammad Chehrazi, Farzin Sadeghi

**Affiliations:** 1 Student Research Committee, Babol University of Medical Sciences, Babol, Iran; 2 Infectious Diseases and Tropical Medicine Research Center, Health Research Institute, Babol University of Medical Sciences, Babol, Iran; 3 School of Dental Medicine, Oral Health Research Center, Babol University of Medical Sciences, Babol, Iran; 4 Department of Biostatistics and Epidemiology, School of Public Health, Babol University of Medical Sciences, Babol, Iran; 5 Cellular and Molecular Biology Research Center, Health Research Institute, Babol University of Medical Sciences, Babol, Iran

**Keywords:** Epstein-Barr Virus, Oral squamous cell carcinoma, Oral lichen planus, Oral irritation fibroma

## Abstract

**Background:**

Epstein-Barr Virus (EBV) is a human oncogenic virus that can lead to cancer in lymphoid and epithelial cells and is one of the hypothesized causes of oral cavity lesions including oral squamous cell carcinoma (OSCC), but the etiological association remains undetermined. The present investigation aimed to explore the EBV presence, viral load, and EBV-encoded small RNA (EBER) sequence variation in tissue samples of patients with OSCC and other oral cavity lesions including oral lichen planus (OLP), and oral irritation fibroma (OIF).

**Methods:**

In total, 88 oral cavity samples (23 with OSCC, 29 with OLP, and 36 with OIF diagnosis) were examined by Real-Time PCR technique and some of them were sequenced.

**Results:**

Viral EBER sequence was detected in 6 out of the 23 OSCC (31.4%), 6 out of the 29 OLP (20.7%), and 3 out of the 36 OIF cases (8.3%). The mean EBV copy number was higher in OSCC samples (1.2 × 10^−2^ ± 1.3 × 10^−2^ copies/cell) compared to OLP (2.2 × 10−^3^ ± 2.6 × 10^−3^ copies/cell) and OIF (2.4 × 10^−4^ ± 2.0 × 10^−4^ copies/cell) samples, although this difference was not statistically significant (P = 0.318). The EBER gene was amplified and sequenced in 5 OSCC, 3 OLP, and 2 OIF samples with high EBV viral load. One OSCC, two OLP, and two OIF isolates showed different nucleotide variations compared with EBV-WT and AG876 prototype sequences: C6834T, C6870T, C6981T, C7085T, C7085G, and C7094T.

**Conclusion:**

In our study the presence of more than one genome copies per tumor cell indicates the possible role of EBV infection in oral cancers. However, more studies should be conducted to clarify the role of EBV in OSCC carcinogenesis.

## Background

Oral squamous cell carcinoma (OSCC) is considered the most prevalent subset (90%) of oral cancer [1]. This malignancy has a survival rate of up to 80% if diagnosed early, compared to a survival rate of 20–30% if detected in late tumor stages [2]. It is the sixth most common malignancy worldwide [3]. The incidence and distribution of OSCC vary in different geographical locations and depends on various factors such as tobacco smoking, alcohol drinking, nutrition, and genetic components [4–6]. To perceive the full spectrum of oral carcinogenesis, the study of oral premalignant lesions (e.g., oral lichen planus) that carry a risk of malignant transformation is necessary. Oral lichen planus (OLP) is a common chronic inflammatory disorder with unknown etiology in which occurs as the result of abnormal T-cell-secreted cytokines in basal epithelial cells and resulting in either epithelial thickening or atrophy [7, 8]. The specific role of human tumor viruses including the Human Papilloma Virus (HPV) and Epstein-Barr Virus (EBV) in the development of OSCC and OLP continues to be a debated topic [9, 10]. Researchers proposed the first evidence for an association between OSCC and infectious agents, especially viruses, about two decades ago [11–13].

Epstein—Barr virus is a widespread human gamma herpes virus with oncogenic potential. It has been implicated in several malignant and benign lesions in the oral cavity and/or head and neck region including Burkitt’s lymphoma (BL), nasopharyngeal carcinoma (NPC), and oral hairy leukoplakia (OHL). The ability of EBV to infect oral and salivary glands epithelium and viral shedding via saliva indicate a possible role of the virus on OSCC pathogenesis. Despite the well-established impact of the EBV on BL and NPC, the influence of EBV in the etiology of OSCC remains uncertain. There have been several reports of EBV genome detection in both cancerous and non-cancerous oral epithelial tissues [14–19]. However, the association between EBV and OSCC remains controversial.

Regarding the role of carcinogenic viruses (e.g., EBV) in the development of cancer, several reports indicate that high viral load being proportional to the risk of viral oncogenesis and the number of viral genome copies per cell is an important indicator [20, 21]. For example, the higher EBV genome copy number increases the risk of Burkitt's lymphoma [22]. In addition, several lines of evidence indicate that certain variants of EBV with distinct polymorphisms in the EBV-encoded small RNA (EBER) region are strongly linked with NPC [23, 24]. Characterization of viral mutations in the EBER region seems important for EBV positive OSCC samples.

Thus, the facts reviewed above encouraged us to explore the presence of EBV genome in tissue samples of 88 patients with oral cavity lesions including OSCC, OLP, and oral irritation fibroma (OIF) and evaluate the positive samples in terms of viral copy number per cell. Moreover, genomic sequences of EBV in the EBER region were compared in 10 EBV positive samples isolated from OSCC, OLP, and OIF groups.

## Methods

### Patients and tissue samples

In this retrospective cross-sectional study, a total of 88 Formalin-fixed paraffin-embedded (FFPE) resection specimens were collected from the archives of the Pathology Department, School of Dentistry, affiliated to Babol University of Medical Sciences between 2015 -2017. All of the samples were subjected to histopathological diagnosis by an experienced pathologist. Out of the 88 FFPE samples, 23 had a histopathologic OSCC diagnosis, 29 and 36 samples had an OLP and an OIF diagnosis, respectively. The demographic and clinical characteristics of patients, including age, gender, anatomical localization of the specimens, and tumor grade were collected from subject's medical records. This study was approved by the Ethical Committee of Babol University of Medical Sciences (IR.MUBABOL.HRI.REC.1398.070), and for all subjects, written informed consent was obtained.

### DNA extraction

Removal of paraffin from FFPE tissue sections was performed according to a previously described procedure [25]. Briefly, 5-μm-thick tissue slices were collected in a sterile nuclease-free microcentrifuge tube (10 sections from each tissue block and approximately 25 mg of FFPE sections). To prevent cross-contamination, a different microtome blade was utilized for each FFPE block. In order to dissolve the paraffin, the FFPE sections were incubated 3 times in 500 μL of xylene for 10 min at 60 °C and were subsequently washed with absolute ethanol. DNA was isolated using the DNA Extraction Mini Kit from Tissue (Yekta TajhizAzma, Tehran, Iran) according to the manufacturer’s instructions. In brief, for tissue digestion and protein removal, 200 μL of tissue lysis buffer and 20 μL of proteinase K (10 mg/mL) were added to each tube. Samples were subsequently incubated at 56 °C overnight. DNA cleanup was done by mini spin column (silica matrix) according to the manufacturer’s instructions. As an extraction negative control, sterile microcentrifuge tubes containing reaction mixtures were processed simultaneously with the tissue samples. The quality and quantity of extracted DNA were evaluated with a nanodrop spectrophotometer (Thermo Fisher Scientific, Wilmington, DE, USA) using the absorption ratio of 260/280 nm.

### EBV quantitative real-time PCR

To detect and measure the amount of EBV viral load, quantitative real-time PCR was performed using a Rotor-Gene Q real-time PCR system (QIAGEN GmbH, Hilden, Germany), through the primer sets and a TaqMan probe specific for the EBV EBER gene according to a previously described procedure [26]. The EBV viral load was determined as the viral DNA copies/ half of the RNase P gene copy (each diploid cell contains two copies of RNase P gene), which normalizes viral copies to the number of cell equivalents as described previously [27]. Construction of plasmids containing cloned target sequences of EBV EBER and human RNase P gene (quantitative standards for real-time PCR) was done by gene synthesis service (Shanghai Gene ray Biotech Co., Ltd). Each reaction consisted of 100 ng of extracted DNA, 12.5 µL YTA qPCR Probe Master Mix (Yekta TajhizAzma, Tehran, Iran), 0.3 µM each primer, and 0.2 µM dual-labeled probe in a 25µL total reaction. In a biological hood, real-time PCR reaction mixes were prepared in a dedicated clean room. Each real-time PCR run included reaction mixtures without DNA template as a non-template control (NTC) and DNA extracted from supernatant of EBV-producing B-cell line (B95-8) as a positive control. To assess the sensitivity of the real-time PCR assay, a standard curve was generated using a tenfold dilution series of EBV EBER plasmid in genomic extracts obtained from EBV-negative FFPE samples.

### Sequencing analysis of the EBV EBER gene

Ten EBV positive cases with high viral load isolated from OSCC, OLP, and OIF groups were subjected to sequence analysis. A 659 bp fragment of the EBER from nucleotide positions 6758–7416 (based on wild type EBV; GenBank accession number NC_007605) was amplified by PCR according to a previously described procedure [23]. The purified PCR products were sent to Bioneer Corporation (Daedeok-gu, Daejeon, South Korea) for dideoxy-DNA sequencing. Sequencing was performed on both strands using the same primers as in PCR. The raw sequencing data were aligned and evaluated using CLC Genomics Workbench (CLC Bio, Aarhus, Denmark). The representative EBV EBER nucleotide sequences reported in this study were deposited in the GenBank with accession numbers MN921212 to MN921221. The maximum-likelihood method was utilized for the construction of a phylogenetic tree of the EBV EBER region (nucleotide positions 6758–7416) for all of the sequences, using MEGA7 software. Statistical significance of the phylogenetic tree was checked by the bootstrap method (1,000 replicates). Besides for out-group, we used a viral sequence other than EBV (Herpes Simplex Virus Type 1).

### Statistical analysis

SPSS version 23 was used to conduct the statistical analysis (SPSS Inc., Chicago, IL, USA). The chi-square analysis was used to analyze differences in proportions between categorical variables. The Kolmogorov–Smirnov test was used to determine if the variables had a normal distribution. We used the independent-samples T-test and the Kruskal–Wallis test for analysis of continuous variables. Statistical significance was described as a P value of less than 0.05.

## Results

### Study patient characteristics

All 88 enrolled subjects (35 men and 53 women, mean age, 51.6 ± 17 years; range, 12–87) were categorized in to three groups: (1) subjects with histopathological confirmed malignant OSCC (n = 23, 13 men and 10 women; mean age, 68.6 ± 13.4 years) (2) those with oral pre-malignant OLP disorder (n = 29, 11 men and 18 women; mean age, 46.7 ± 12 years) and (3) subjects with non-neoplastic OIF (n = 36, 11 men and 25 women; mean age, 44.1 ± 14.9). The demographic and clinical characteristics of the participants are displayed in Table 1. The patient's samples had been taken from different sites in the oral cavity including buccal mucosa (44.3%), gingiva & alveolar mucosa (14.8%), tongue (12.5%), labial mucosa (11.4%), palate (4.5%), and floor of mouth (1.1%). The anatomical localization of 10 samples was unknown.

**Table 1:** Summary of demographic, clinical and virologic characteristics of the study population

Variables	OSCC	OLP	OIF	P value
Mean age ± SD	68.6 ± 13.4	46.7 ± 12	44.1 ± 14.9	0.001*
Gender (male/female)	13/10 (56.5%/43.5%)	11/18 (37.9%/62.1%)	11/25 (30.5%/69.5%)	0.1
Anatomical localization of Lesions				0.04*
Buccal Mucosa	6 (15.4%)	20 (51.3%)	13 (33.3%)	
Floor of Mouth	1 (100%)	0	0	
Gingiva & alveolar mucosa	8 (61.5%)	2 (15.5%)	3 (23%)	
Labial mucosa	2 (20%)	2 (20%)	6 (60%)	
Palate	1 (25%)	1 (25%)	2 (50%)	
Tongue	4 (36.4%)	2 (18.2%)	5 (45.5%)	
Tumor grade				
I	11 (47.8%)	–	–	
II	7 (30.4%)	–	–	
III	3 (13.1%)	–	–	
Unknown	2 (8.7%)	–	–	
EBV (positive/negative)	6/17 (26.1%/73.9%)	6/23 (20.7%/79.3%)	3/33 (8.4%/91.6%)	0.18
Mean EBV Viral Load copies per cell	1.2 × 10^−2^ ± 1.3 × 10^−2^	2.2 × 10^−3^ ± 2.6 × 10^−3^	2.4 × 10^−4^ ± 2.0 × 10^−4^	0.318

*statistical significance was described as a P < 0.05

### Detection and quantitation of EBV

DNA quality and integrity of all included samples were verified by successful amplification of the human RNase P gene. Samples with cycle threshold (Ct) values ≥ 30 for human RNase P were considered to have inadequate DNA quality and therefore were re-extracted until RNase P amplification could be achieved with Ct values < 30. The results from EBV detection revealed the presence of the viral EBER gene in a total of 15 (17%) out of the 88 tested samples (4 men and 11 women; mean age, 55.7 ± 15.9 years). No significant differences were found between EBV positivity and gender (P = 0.386). Also, no statistically significant difference between patients’ mean age and EBV positivity was seen (P = 0.570). Of the 23 OSCCs, EBV DNA was detected in 6 tumors (26.1%). In detail, out of six OSCC positive samples, EBV DNA was detected in three (50%) gingiva & alveolar mucosa, one (16.6%) of buccal mucosa, one (16.6%) of the floor of the mouth. The anatomical localization of one EBV positive OSCC sample was unknown. Regarding the grade of tumor in the OSCC group, EBV genome was detected in 36.4% (4/11) of grade I, and 14.3% (1/7) of grade II tumors. The grade of the tumor was unknown in one EBV positive OSCC. Of the 29 OLPs, EBV DNA was detected in 6 samples (20.7%). In the OLP group, EBV DNA was detected in four (66.6%) of buccal mucosa, one (16.6%) of labial mucosa, and one (16.6%) of the palate. Epstein—Barr virus DNA was detected in oral specimens of only 3 out of the 36 OIF cases (8.3%), specifically, two (5.5%) gingiva & alveolar mucosa, and one (16.6%) of the buccal mucosa. There was no significant difference in EBV positivity between the three study groups (OSCC, OLP, and OIF) (P = 0.18) (Table 1).

**Fig. 1:**
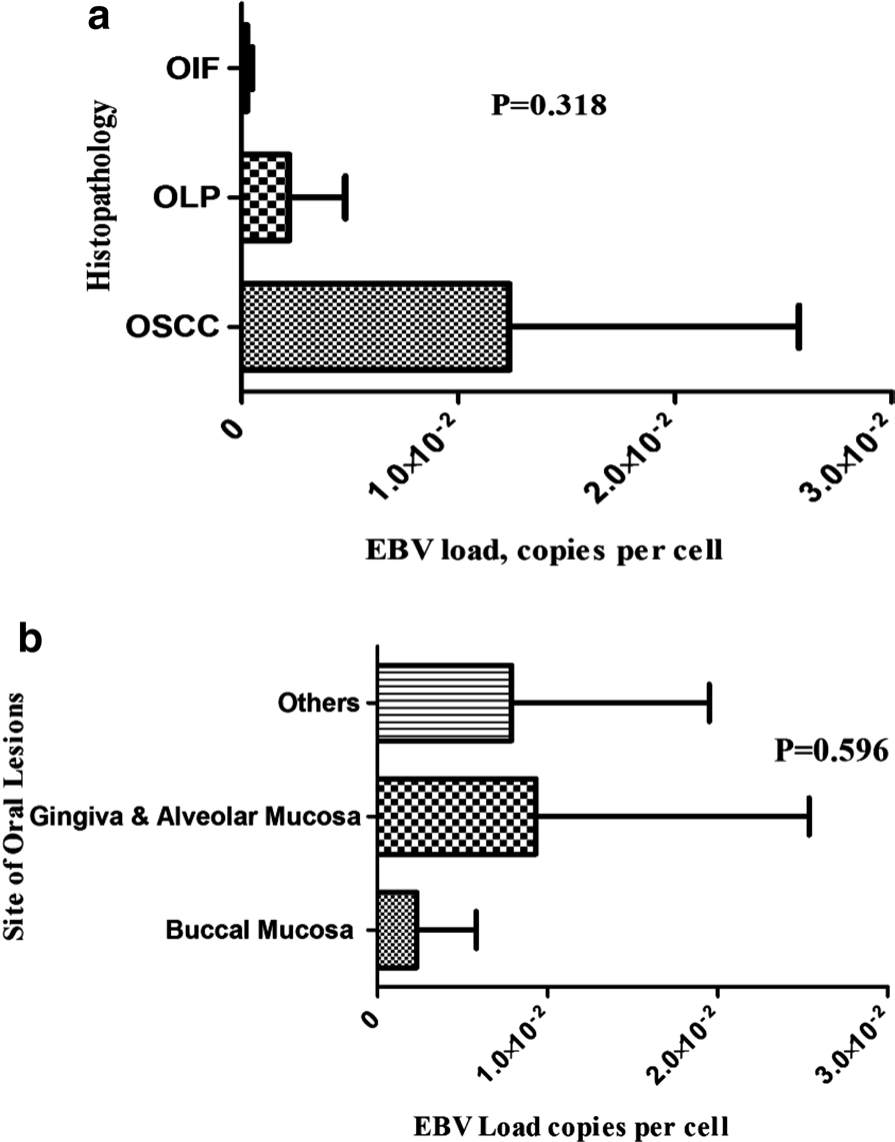
The mean EBV DNA load in different histopathological groups (**A**), and various site of lesions in oral cavity (**B**)

The EBV DNA load was determined as the viral copy number per cell using a proven single-copy gene, human RNase P. In EBV positive samples, the mean EBV copy number was higher in OSCC samples (1.2 × 10^−2^ ± 1.3 × 10^−2^ copies/cell) compared to OLP (2.2 × 10^−3^ ± 2.6 × 10^−3^ copies/cell) and OIF (2.4 × 10^−4^ ± 2.0 × 10^−4^ copies/cell) samples, although this difference was not statistically significant (P = 0.318) (Fig. 1A). In terms of the various site of lesions in the oral cavity the mean EBV copy number in the buccal mucosa, gingiva & alveolar mucosa, and other sites were 2.3 × 10^−3^ ± 3.5 × 10^−3^, 9.3 × 10^−3^ ± 1.6 × 10^−2^, and 7.9 × 10^−3^ ± 1.1 × 10^−2^ copies/cell, respectively. There was no statistically significant difference between the various site of lesions and EBV DNA load (P = 0.596) (Fig. 1B). Regarding the grade of OSCC tumor the mean EBV copy number in grade I tumors was 1.7 × 10^−2^ ± 1.5 × 10^−2^, and a very low level of EBV DNA (7.0 × 10^−5^ copies/cell) was found in only 1 EBV positive grade II tumor.

### EBER sequence variations

**Fig. 2:**
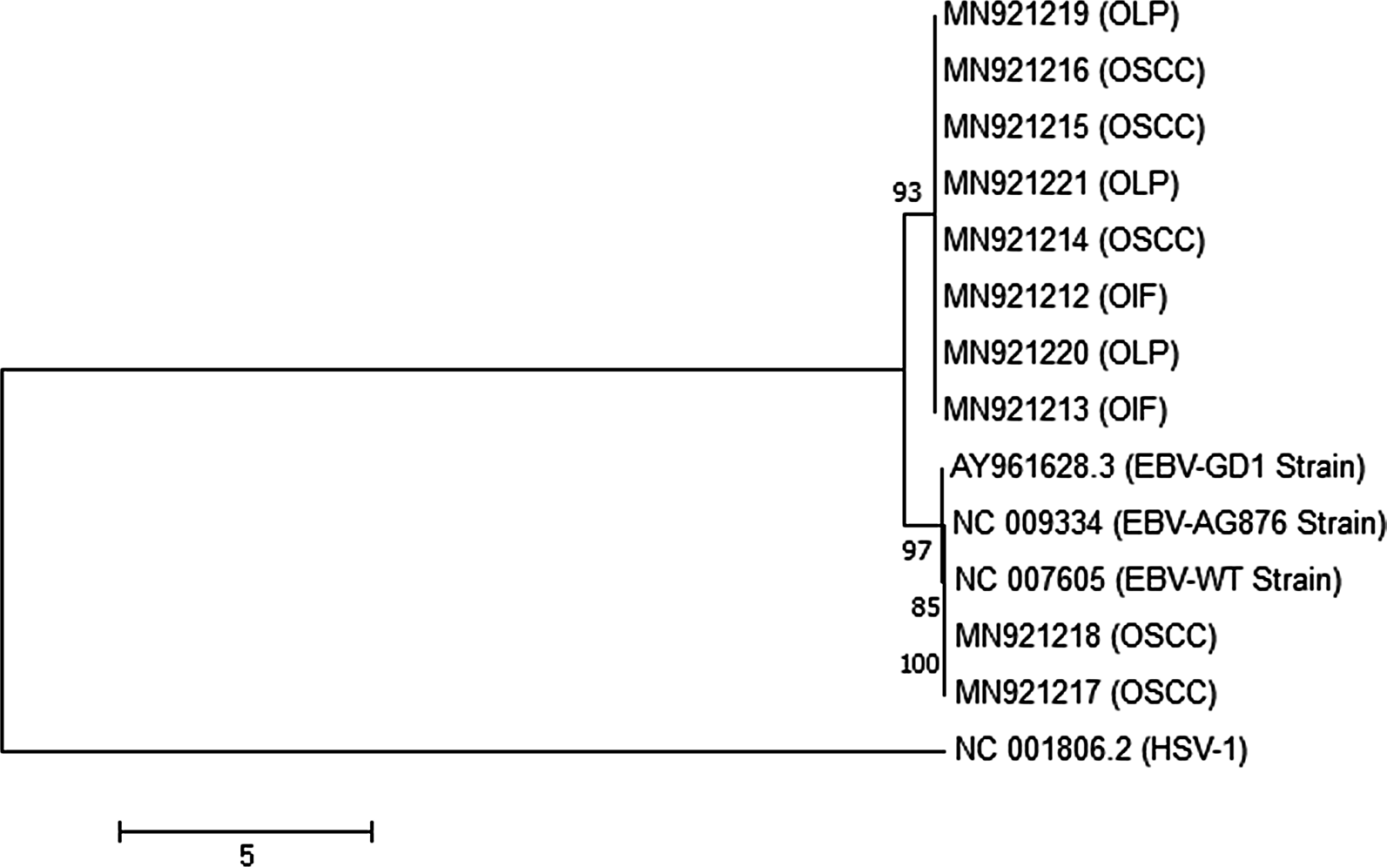
Phylogenetic tree for sequences of ten selected EBV positive patients in the EBER region (nucleotide positions 6758–7416) with MEGA (version 7.0) software. The patient group (OSCC, OLP and OIF), and accession number are listed. The evolutionary history was inferred by using the Maximum Likelihood method based on the Tamura-Nei model [55]. The tree with the highest log likelihood (-2588.74) is shown

The EBER gene was amplified and sequenced in 5 OSCC, 3 OLP, and 2 OIF samples with high EBV viral load. Comparison of nucleotide sequences of the coding region of EBER2 (6956–7128) as well as non-coding region between EBER1 and EBER2 (6796–6955) with prototype EBV-WT reference sequence was performed. The sequence variations are shown in Table 2. Mutations at the seven nucleotides have been regarded as the type-specific mutations (6808, 6884, 6886, 6911, 6927, 6944, and 7123), and sequences identical to EBV-WT at the seven positions are classified as type 1 EBER sequences, whereas those identical to AG876 at the seven positions are classified as type 2 EBER sequences [28, 29]. In the present study, all the 10 isolates are classified as type 1 EBER sequence according to the similarity in the aforementioned seven nucleotide positions. One OSCC, two OLP, and two OIF isolates showed different nucleotide variations compared with EBV-WT and AG876 prototype sequences: C6834T, C6870T, C6981T, C7085T, C7085G, and C7094T. The 10 sequences of the EBER region (nucleotide positions 6758–7416) from all of the patients and the 3 EBER sequences of reported EBV genomes (EBV-WT, AG876, and GD1 strain) were used to make a phylogenetic tree (Fig. 2). The phylogenetic tree is labeled with patient group (OSCC, OLP, and OIF), and accession number. No clustering according to the patient group was observed among the sequences.

**Table 2:** Nucleotide sequence variations in the EBER region of 10 samples with OIF, OLP, and OSCC

											Intergenic Region (6796–6955)		EBER-2 (6956–7128)
Oral Lesions	Position	6808	6834	6870	6884	6886	6911	6927	6944	6981	7085	7094	7123
	EBV-WT (Reference)	T	C	C	G	T	A	G	G	C	C	C	A
	AG876	A	–	–	A	G	G	A	A	–	–	–	G
OIF	MN921213.1	–	T	T	–	–	–	–	–	–	T	T	–
OIF	MN921212.1	–	T	–	–	–	–	–	–	–	G	–	–
OLP	MN921219.1	–	–	–	–	–	–	–	–	–	–	–	–
OLP	MN921220.1	–	–	–	–	–	–	–	–	T	T	T	–
OLP	MN921221.1	–	T	–	–	–	–	–	–	–	G	–	–
OSCC	MN921214.1	–	T	–	–	–	–	–	–	–	G	–	–
OSCC	MN921216.1	–	–	–	–	–	–	–	–	–	–	–	–
OSCC	MN921217.1	–	––	–	–	–	–	–	–	–	–	–	–
OSCC	MN921218.1	–	–	–	–	–	–	–	–	–	–	–	–
OSCC	MN921215.1	–	–	–	–	–	–	–	–	–	–	–	–

Only nucleotides different from EBV-WT are indicated. The changes in coding region of EBER2 and non-coding region between EBER1 and EBER2 are separated by dark line and the positions are shown in the top. The nucleotide positions of identified changes are indicated vertically across the top. The absence of variations is showed with dashes. Previously published sequence of AG876 was taken from Lin et al. [28] and Dolan et al. [54]

## Discussion

Oral cancer is the sixth most frequent cancer in the human population, with OSCC being the most common type [30]. Oral squamous cell carcinoma can be caused by a variety of risk factors, including oncogenic viruses [31]. In different parts of the world, various EBV prevalence rate on OSCC samples has been reported [14, 15, 32–34]. Different research groups have different perspectives on the role of EBV in OSCC. This inconsistency may be as a result of using different methods in studies, geographic and ethnic variations and also the effects of the other risk factors.

In several studies with no evidence of EBV detection in OSCC samples, the "hit and run" theory was proposed. According to this theory, viral DNA is only necessary for malignancy induction and then vanishes during the host's uncontrolled cell cycles [35]. In some studies, EBV genomic sequences has been isolated from OSCC samples using PCR technique [18, 36]. In addition, EBV EBER was found to be overexpressed in extreme epithelial dysplasia and linked to carcinogenesis [37]. In this way, we used the EBER gene to evaluate the presence of EBV in the OSCC samples. In the present study, the patient's samples were taken from different sites in the oral cavity. The results from EBV detection revealed the existence of the viral EBER gene in 17% of samples (31.4% of OSCC, 20.7% of OLP and 8.3% of OIF). According to an investigation, EBV infection in OSCCs was detected by PCR in 52.8 percent of cases and by in-situ hybridization (ISH) EBER was found in 27.5 percent of cases [38]. In another study, the researchers found positivity for EBV infection in all of 36 (100%) OSCCs, 7 out of 9 (77.8%) pre-cancerous lesions, and 1of 12 (8.3%) normal mucosa [39], which show the high frequency of EBV infection in OSCC samples. A Taiwanese research team using an EBV genomic microarray found a high rate of EBV infection (82.5%) in biopsy specimens from 57 OSCC cases [40]. Reporting the high prevalence of EBV infection in their research can be as a result of using fresh tissue samples and higher technical sensitivity. The other two studies which evaluated FFPE samples with PCR method, also declared that the EBV prevalence was higher in OSCC in comparison with healthy individuals [18, 41].

In contrast, Lamaroon and colleagues in a study conducted in Thailand didn’t consider EBV as an OSCC risk factor since the EBER gene was not detectable in their 24 cases of OSCC [42]. Similarly, Kerishnan et al. revealed that their results didn’t demonstrate any association between EBV infection and OSCC [43]. About the location of malignancies, we didn’t see any statistically significant difference between the various sites of lesions and EBV DNA detection. Our findings were consistent with two other studies [15, 44]. Furthermore, no statistically significant difference between patients’ mean age and EBV positivity was seen in our findings. Comparable to our results there was also no link between EBV viral load and patient age in positive cases in Saravani et al. investigation [45].

The presence of more than one genome copies per tumor cell, which is generally seen in BL and NPC, suggests direct EBV oncogenic role. In the current investigation, in accordance with Goldenberg et al. [46], less than one EBV genome per cell has been detected in OSCC samples. Low copy numbers of virus genome per tumor cell, suggests persistence of EBV as a passenger virus in the oral cavity without obvious pathological consequence. However, several studies have demonstrated substantially higher levels of EBV genome in OSCC compared non-cancerous tissues [47–50].

In this study, ten EBV positive cases with high viral load isolated from OSCC, OLP, and OIF groups were subjected to EBV mutation analysis of spacer and EBER2 sequences to gain a true picture of EBER variant polymorphisms. One OSCC, two OLP, and two OIF isolates showed different nucleotide variations compared with EBV-WT and AG876 prototype sequences. However, no consensus mutations were found among the isolates regarding three main EBER variants (EB-6 m, EB-8 m, and EB-10 m). Three significant EBER gene variations denoted as EB-6 m, EB-8 m, and EB-10 m, were found in a study on northern Chinese isolates from the NPC non-endemic area, which EB-8 m and EB-10 m, had six common mutations at the EBER2 coding region [51]. EBV-encoded small RNA gene polymorphism has received little research because it is widely assumed that the coding sequences of EBER1 and EBER2 are conserved between EBV strains [28, 29]. However, this viral gene has a small amount of sequence variation, which has been related to the EBV Type 1/Type 2 status [29, 52]. In the present study, all the 10 isolates are classified as EBV Type 1, based on the similarity to EBV-WT at the EBER sequence. Sequence analysis of EBV strains B95-8, P3HR-1, and AG876 revealed two patterns of EBER variants, B95-8 and AG876/P3HR-1, with the majority of type-specific mutations occurring in the 161-bp noncoding spacer region [29]. In another study by Xiaoshi et al., EBERs from NPC tissue possessed the basic characteristics of type 1 EBV, as well as 8 base-pair substitutions and 2 base pair deletions in EBER-2 [53].

Taken together, current study had some limitations including, small sample size, lack of fresh or non-paraffin samples, and no evaluation of non-cancerous tumor margin.

## Conclusion

This study found that EBV is more common in oral lesions like OSCC and OLP than in OIF with a low copy number of viral genomes. The presence of EBV in our samples can show the indirect role of this virus in oral disease pathogenesis, that they use a "hit and run" process, or that they are only involved in a small percentage of cases. However, with the limitation of the findings, many issues about the molecular pathways underlying EBV infection-induced carcinogenesis remain unanswered, so prospective follow-up studies are needed to investigate the mechanisms by which EBV promotes carcinogenesis. Also, epidemiological and molecular investigations, especially on oral fresh biopsy samples and in case–control setting should be done to ascertain the role of EBV in oral carcinogenesis.

## Data Availability

The datasets used and/or analysed during the current study are available from the corresponding author on reasonable request.

## Abbreviations

EBV: Epstein-Barr Virus
OSCC: Oral squamous cell carcinoma
EBER: EBV-encoded small RNA
OLP: Oral lichen planus
OIF: Oral irritation fibroma
HPV: Human Papilloma Virus
BL: Burkitt’s lymphoma
NPC: Nasopharyngeal carcinoma
OHL: Oral hairy leukoplakia
FFPE: Formalin-fixed paraffin-embedded
NTC: Non-template control
CT: Cycle threshold
WT: Wild type
